# Seronegative Autoimmune Encephalitis: A Case Report and Literature Review

**DOI:** 10.7759/cureus.105381

**Published:** 2026-03-17

**Authors:** Carmen Fierro, Andrea Laurienzo, Vincenzo Cuzzone

**Affiliations:** 1 Anesthesia and Critical Care, A. Cardarelli Hospital, Campobasso, ITA; 2 Neurology, A. Cardarelli Hospital, Campobasso, ITA

**Keywords:** autoimmune encephalitis, csf, immunotherapy, mri, seronegative encephalitis

## Abstract

Autoimmune encephalitis is an inflammatory disorder of the central nervous system frequently associated with antibodies directed against neuronal surface or intracellular antigens. However, a substantial subset of patients remains seronegative, posing diagnostic challenges.

We report the case of a 65-year-old man who presented to the emergency department with acute confusion, prosopagnosia, psychomotor agitation, and generalized tonic-clonic seizures, followed by coma requiring intubation and intensive care admission. Extensive microbiological findings, including CSF PCR for herpes simplex virus (HSV) and varicella-zoster virus (VZV) and CSF cultures, as well as a comprehensive autoimmune encephalitis panel in serum and CSF (anti-NMDA, anti-AMPA, anti-GABA, anti-LGI1, anti-CASPR2, anti-DPPX), were all negative. Brain magnetic resonance imaging demonstrated faint diffuse cortical hyperintensity on diffusion-weighted imaging, more pronounced in the temporo-parietal regions, without contrast enhancement. Electroencephalography revealed diffuse epileptiform activity. Based on clinical presentation, neuroimaging findings, exclusion of alternative etiologies, and electroencephalographic abnormalities, a diagnosis of probable seronegative autoimmune encephalitis was established. The patient was treated with high-dose intravenous methylprednisolone followed by intravenous immunoglobulins, resulting in rapid neurological improvement and complete recovery (modified Rankin Scale (mRS) score of 1 upon discharge from the hospital).

This case highlights the importance of clinical judgment in suspected autoimmune encephalitis, even in the absence of detectable neuronal antibodies, and underscores the potential for favorable outcomes with early immunotherapy.

## Introduction

Encephalitis is an inflammatory disorder of the central nervous system. When microbiological investigations are negative, encephalitis is often autoimmune in origin and is associated with autoantibodies targeting neuronal antigens [1,2]. In a subset of patients, serum and cerebrospinal fluid (CSF) antibody panels may be negative, posing diagnostic and therapeutic challenges [3,4].

Autoimmune encephalitis encompasses a heterogeneous group of immune-mediated inflammatory disorders affecting the brain. The identification of neuronal autoantibodies has significantly improved diagnostic accuracy; however, up to up to 50% of cases [5] may be seronegative despite fulfilling clinical criteria for autoimmune encephalitis.

In such cases, diagnosis relies on clinical presentation, neuroimaging, CSF analysis, electroencephalography findings, exclusion of alternative causes, and response to immunotherapy [6]. We report a case of probable seronegative autoimmune encephalitis fulfilling Graus criteria and responding to early immunotherapy. Prompt recognition is essential, as early immunomodulatory treatment has been associated with improved neurological outcomes.

## Case presentation

A previously healthy 65-year-old man presented to the emergency department with an acute onset of disorientation in time and space, prosopagnosia, and psychomotor agitation lasting several hours. His past medical history was characterized only by hypertension on treatment and active smoking. No antiplatelet and anticoagulant therapy or allergies reported. Shortly after arrival, he developed generalized tonic-clonic seizures followed by loss of consciousness progressing to coma. On examination after the seizure, his Glasgow Coma Scale score was 4 (E1, V1, M2), and his body temperature was 38.2°C. The patient was intubated and transferred to the intensive care unit (Table 1).

**Table 1:** Clinical timeline of the patient’s presentation, diagnostic workup, and management. The table summarizes the chronological sequence of the patient’s clinical course, including the onset of neurological symptoms, key diagnostic investigations, and therapeutic interventions from admission to the intensive care unit through the subsequent diagnostic work-up. Laboratory and CSF findings, microbiological testing, and autoimmune encephalitis screening are reported to illustrate the stepwise clinical evaluation and management. GCS: Glasgow Coma Scale; CSF: cerebrospinal fluid; PCR: polymerase chain reaction

Time point	Clinical events	Diagnostic findings	Management
Day 0: Symptom onset	Acute onset of disorientation in time and space, prosopagnosia, and psychomotor agitation lasting several hours	-	-
Day 0: Emergency Department admission	Development of generalized tonic-clonic seizures followed by loss of consciousness progressing to coma; body temperature 38.2°C; GCS 4	Initial blood tests: moderate leukocytosis; normal CRP; elevated transaminases, creatine kinase, and lactate dehydrogenase	Patient intubated and transferred to the intensive care unit
Days 0-1	Persistent coma after seizure	CSF: clear; 1 mononuclear cell/µL; protein 121 mg/dL; normal glucose	Supportive care in the ICU
Day 1	Infectious causes investigated	CSF PCR negative for herpes simplex virus and varicella-zoster virus; CSF cultures negative	Empirical/targeted management according to clinical suspicion
Days 1-2	Evaluation for autoimmune encephalitis	Autoimmune encephalitis panel in serum and CSF negative (anti-NMDA, anti-AMPA, anti-GABA, anti-LGI1, anti-CASPR2, anti-DPPX antibodies)	Continued monitoring and supportive management

Initial laboratory testing showed moderate leukocytosis (WBC 19.23 x 10^3^/uL), normal C-reactive protein (3.20 mg/L), and elevated transaminases, creatine kinase, and lactate dehydrogenase levels. CSF was clear, with one mononuclear cell per microliter, protein concentration of 121 mg/dL (normal), and normal glucose levels (Table 2). Immunologic tests showed IgA 1.42 g/L, IgG 9.32 g/L, and IgM 1.32 g/L. Onconeural markers were determined: all were negative (anti-amphiphisin, anti-CV2, anti-PNMA2 (Ma2/Ta), anti-Ri (ANNA-2), anti-Yo (PCA-1), anti-Hu (ANNA-1), anti-recoverina, anti-SOX1, anti-titin, anti-Zic4, anti-GAD65, anti-Tr (DNER). Microbiological investigations, including CSF polymerase chain reaction testing for herpes simplex virus and varicella-zoster virus, were negative. CSF cultures showed no microbial growth. A comprehensive autoimmune encephalitis panel in both serum and CSF was negative for anti-NMDA, anti-AMPA, anti-GABA, anti-LGI1, anti-CASPR2, and anti-DPPX antibodies.

**Table 2:** Laboratory and CSF analysis. CBC: complete blood count; CRP: C-reactive protein; CSF: cerebrospinal fluid; PCR: polymerase chain reaction; AST: aspartate aminotransferase; ALT: alanine aminotransferase; GOT: glutamic oxaloacetic transaminase; GPT: glutamic pyruvic transaminase; CK: creatine kinase; LDH: lactate dehydrogenase; HSV: herpes simplex virus; VZV: varicella-zoster virus; EBV: Epstein-Barr virus; CMV: cytomegalovirus; HHV: human herpesvirus

Test	Result	Units
CBC	Moderate leukocytosis	-
CRP	initially normal (3.20)	mg/L
Transaminase, creatine kinase, and LDH	GOT 67, GPT 91, CK 4, elevated	IU/L
CSF appearance	Clear	-
CSF cell	1 (mononuclear)	cells/µL
CSF protein	121 (normal)	mg/dL
CSF glucose	67 (normal)	mg/dL
CSF/serum autoimmune encephalitis panel	Negative for anti-NMDA, anti-AMPA, anti-GABA, anti-LGI1, anti-CASPR2, and anti-DPPX	-
CSF PCR (HSV, VZV, EBV, CMV, adenovirus, HSV-1, HSV-2, HHV-6, HHV-7, Parvovirus B19, and enterovirus)	Negative	-
CSF culture	Negative for microbial growth	-

**Figure 1:**
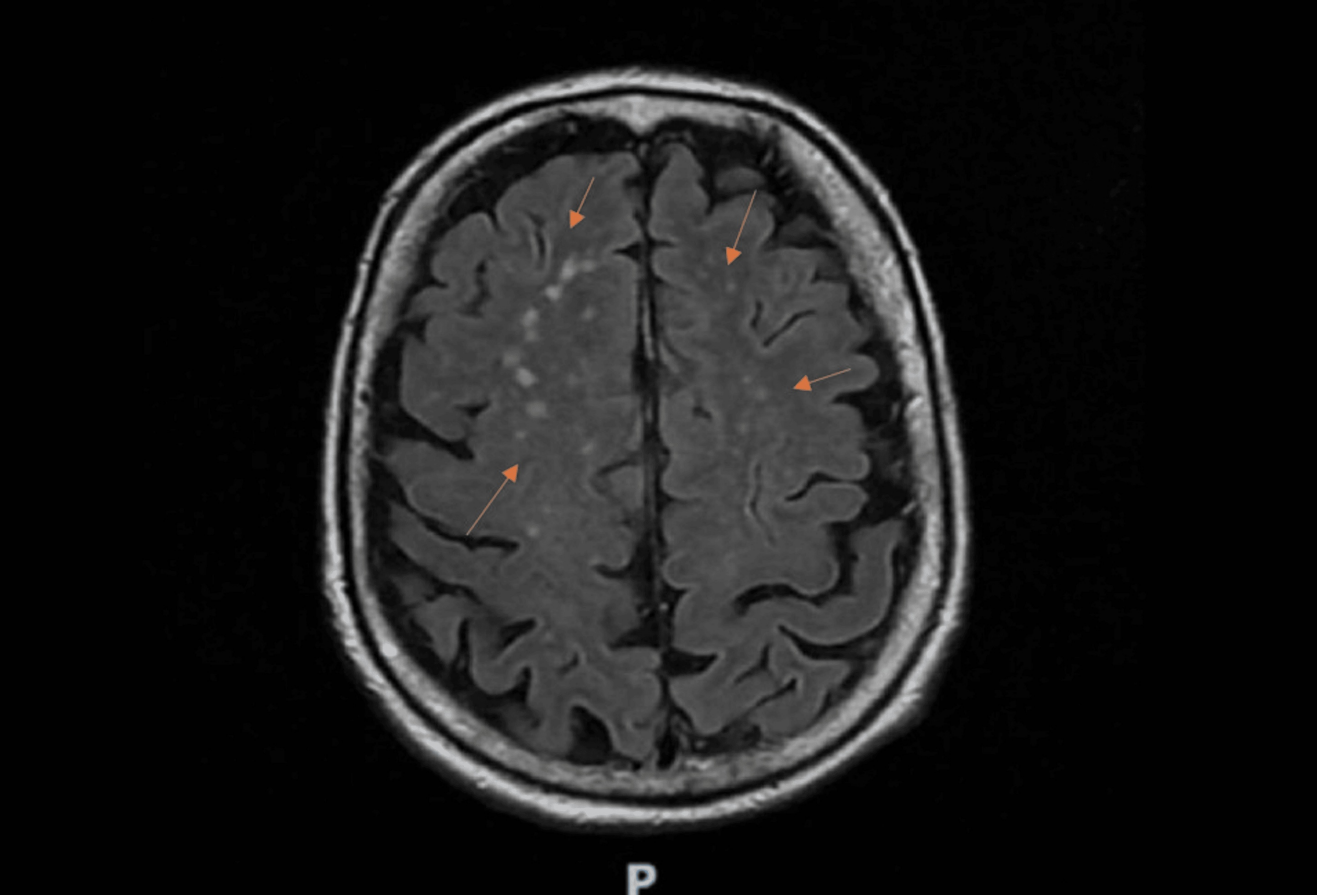
Axial T2 fluid-attenuated inversion recovery (FLAIR) magnetic resonance imaging (MRI) demonstrating faint diffuse cortical hyperintensity, more pronounced in the temporoparietal regions (arrows).

**Figure 2:**
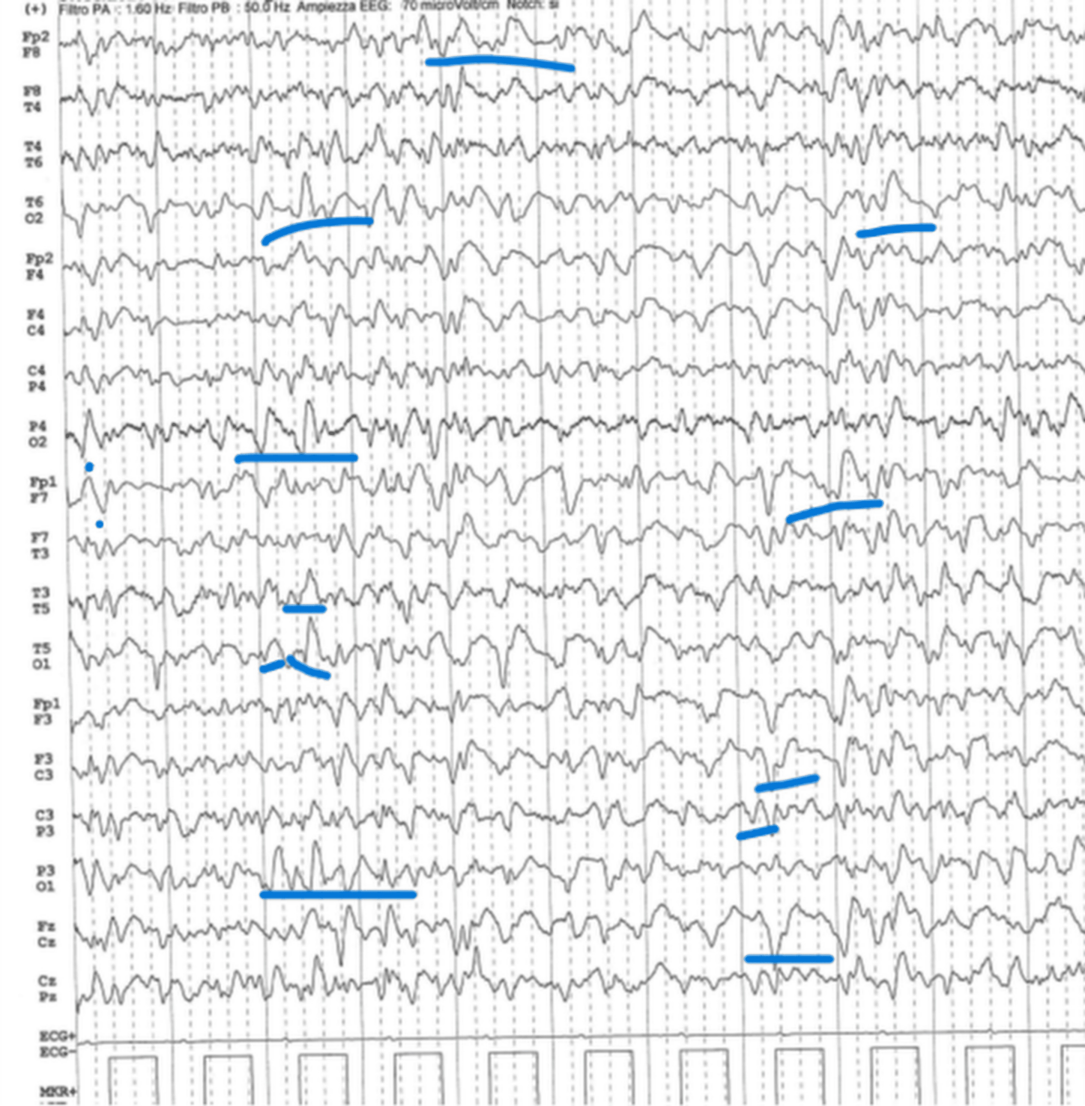
Electroencephalography (EEG) demonstrating diffuse sporadic spike-wave activity.

Cerebral computed tomography angiography was unremarkable. Brain magnetic resonance imaging (MRI) with axial T2 fluid-attenuated inversion recovery (FLAIR) sequences revealed faint diffuse cortical hyperintensity on diffusion-weighted imaging (DWI), more evident in the temporoparietal regions, without pathological contrast enhancement (Figure 1). However, apparent diffusion coefficient (ADC) restriction was present, helping differentiate cytotoxic from inflammatory edema. Electroencephalography performed during sedation weaning demonstrated diffuse sporadic spike-wave activity (Figure 2).

Infectious encephalitis was considered unlikely due to negative microbiological testing and normal CSF glucose levels. Paraneoplastic etiologies were excluded through whole-body computed tomography and negative onconeural antibody testing. Based on the acute neuropsychiatric presentation, MRI abnormalities, electroencephalographic findings, exclusion of alternative causes, and clinical course, a diagnosis of probable seronegative autoimmune encephalitis was established.

Empiric antiviral and antibiotic therapy with acyclovir (10 days long) and ceftriaxone was discontinued after negative infectious workup results. The PCR dosage was repeated approximately every three days. Immunotherapy was initiated with intravenous methylprednisolone at a dose of one gram daily for five consecutive days, followed by intravenous immunoglobulin therapy. Maintenance treatment with oral prednisone was subsequently introduced. Tuberculosis is relatively uncommon in Italy, which is classified by the World Health Organization as a low‑incidence country for tuberculosis with fewer than 10 cases per 100,000 population annually; for example, recent surveillance reports indicate an incidence of approximately 4-6 cases per 100,000 in the Italian population (WHO, Global tuberculosis reports, 1997 to 2025). Tubercular central nervous system involvement was considered in the differential diagnosis; however, it was ruled out in this case based on negative CSF acid‑fast bacilli staining and *Mycobacterium tuberculosis* PCR, the absence of typical CSF features (e.g., markedly elevated protein with low glucose and significant pleocytosis), and lack of neuroimaging evidence suggestive of tuberculous meningitis or focal tuberculoma. These findings support the exclusion of tubercular pathology in the diagnostic work‑up. The patient demonstrated marked neurological improvement within 10 days, with complete seizure control achieved using levetiracetam. Neuropsychological recovery was complete within three weeks (modified Rankin Scale (mRS) score of 1 at discharge), and the patient was discharged in a stable clinical condition.

## Discussion

This case illustrates the diagnostic complexity of seronegative autoimmune meningoencephalitis. Although antibody detection provides important diagnostic confirmation, the absence of identifiable autoantibodies does not exclude the disorder. A significant proportion of patients with clinical features consistent with autoimmune encephalitis may remain antibody-negative, particularly in the early stages of disease or when currently unidentified antibodies are involved [5].

The clinical features observed in this patient are consistent with the criteria for possible autoimmune encephalitis proposed by Graus et al. [5]. The acute onset of neuropsychiatric symptoms, the presence of seizures, MRI abnormalities, electroencephalographic changes, and the exclusion of infectious and neoplastic etiologies supported the diagnosis. CSF findings were only mildly abnormal, which further emphasizes that inflammatory markers may be subtle in some cases.

Early initiation of immunotherapy is associated with improved neurological outcomes, including in seronegative cases [7]. These conditions often show highly distinctive cognitive, seizure, and movement disorder phenotypes, making them clinically recognizable [8]. Corticosteroids alone or combined with other agents (intravenous IG or plasmapheresis) were selected as a first-line therapy by 84% of responders for patients with a general presentation [8]. In this patient, the rapid and sustained clinical improvement following high-dose corticosteroids and intravenous immunoglobulins further supported the presumed immune-mediated pathogenesis. Recognition of seronegative autoimmune encephalitis, therefore, requires a high index of clinical suspicion and a multidisciplinary diagnostic approach integrating clinical, radiological, and electrophysiological data.

## Conclusions

Seronegative autoimmune encephalitis should be considered in patients presenting with acute neuropsychiatric symptoms, seizures, and compatible neuroimaging or electroencephalographic findings when infectious and neoplastic causes have been reasonably excluded. This case highlights the importance of a structured diagnostic approach integrating clinical evaluation, neuroimaging, CSF analysis, and electroencephalography, even when serum and CSF antibody testing are negative. Early empiric immunotherapy may be associated with improved clinical outcomes in selected patients with suspected autoimmune encephalitis and should be considered when the clinical suspicion is high.
